# Obituary

**Published:** 1894-05

**Authors:** 


### Obituary.

Dr. John H. Rauch, of Chicago, the distinguished sanitarian and publicist, was found dead in bed at the residence of his brother, Cyrus G. Rauch, of Lebanon, Pa., March 25, 1894. Dr. Rauch had not been in robust health for some time, and, worn and weary from long continued labor, a few months ago sought rest in his old home where he was born and reared. He graduated from the University of Pennsylvania in 1850, and afterward located at Burlington, Iowa. He entered the civil war as brigade surgeon, and finally became medical director of the Department of the Gulf. After the war he settled in Chicago, and was elected to the faculty in Rush Medical College. He became president, and finally secretary, of the Illinois State Board of Health, and rendered active service in epidemics of yellow fever and cholera. He was a member of the Military Order of the Loyal Legion, chairman of the section of state medicine in the American Medical Association, and one of the trustees of the Association Journal.

Professor Charles Edouard Brown-Sèquard, M. D., the eminent physician, physiologist and scientist, died in Paris, April 2, 1894. He was born in the island of Mauritius in 1818. He took the degree of M. D. in the Paris School of Medicine in 1846, and he delivered a series of lectures before the Royal College of Physicians and Surgeons, in London, in 1858. He took up his residence in the United States in 1864, and was appointed professor of physiology and pathology of the nervous system at Harvard. He returned to France in 1869, when he was appointed professor of experimental physiology in the School of Medicine in Paris, and, in 1878, he succeeded Claude Bernard in the chair of experimental medicine in the College of France. He has been a frequent contributor to the literature of medicine, and his services often have been in demand as a consultant in diseases of the nervous system.

---

Dr. Corydon L. Ford, for forty years Professor of Anatomy in the University of Michigan, died at Ann Arbor, April 14, 1894, in the eighty-second year of his age. Dr. Ford was the first demonstrator of anatomy in the medical department of the University of Buffalo, and, as such, will be well remembered by the students of that period. Later he became one of the most eminent and successful teachers of anatomy in the United States, and pursued his labors with activity until within a few days of his death.

---

Mrs. Rebecca Loop, wife of Dr. D. D. Loop, died at her home in North East, Pa., March 14, 1894, aged sixty-six years. Mrs. Loop was a woman of great usefulness in the community in which she lived, bearing her full share of all the demands of hospitality, charity, church work, and taking great interest in the prosperity of the people among whom she was a beloved and devoted character. Dr. Loop is entitled to, and will receive the sympathy of, a large circle of professional friends throughout the country.

